# Catalytic and Spectroscopic Properties of the Halotolerant Soluble Methane Monooxygenase Reductase from *Methylomonas methanica* MC09

**DOI:** 10.1002/cbic.202100592

**Published:** 2022-01-13

**Authors:** Elisabeth Lettau, Domenic Zill, Marta Späth, Christian Lorent, Praveen K. Singh, Lars Lauterbach

**Affiliations:** ^1^ Technische Universität Berlin Institute of Chemistry Straße des 17. Juni 135 10437 Berlin Germany; ^2^ RWTH Aachen University iAMB – Institute of Applied Microbiology Worringer Weg 1 52074 Aachen Germany

**Keywords:** biocatalysis, enzyme kinetics, methane, NADH reductase, soluble methane monooxygenases

## Abstract

The soluble methane monooxygenase receives electrons from NADH via its reductase MmoC for oxidation of methane, which is itself an attractive C1 building block for a future bioeconomy. Herein, we present biochemical and spectroscopic insights into the reductase from the marine methanotroph *Methylomonas methanica* MC09. The presence of a flavin adenine dinucleotide (FAD) and [2Fe2S] cluster as its prosthetic group were revealed by reconstitution experiments, iron determination and electron paramagnetic resonance spectroscopy. As a true halotolerant enzyme, MmoC still showed 50 % of its specific activity at 2 M NaCl. We show that MmoC produces only trace amounts of superoxide, but mainly hydrogen peroxide during uncoupled turnover reactions. The characterization of a highly active reductase is an important step for future biotechnological applications of a halotolerant sMMO.

## Graphical abstract

**The reductase module** of the soluble methane monooxygenase from *Methylomonas methanica* MC09 is catalytically active for NADH oxidation even at high salt concentrations. Spectroscopic analyses revealed one FeS cluster and FAD. During uncoupled turnover reactions, moderate levels of hydrogen peroxide were released. The characterization of this reductase is an important step for future biotechnological applications of a halotolerant sMMO.

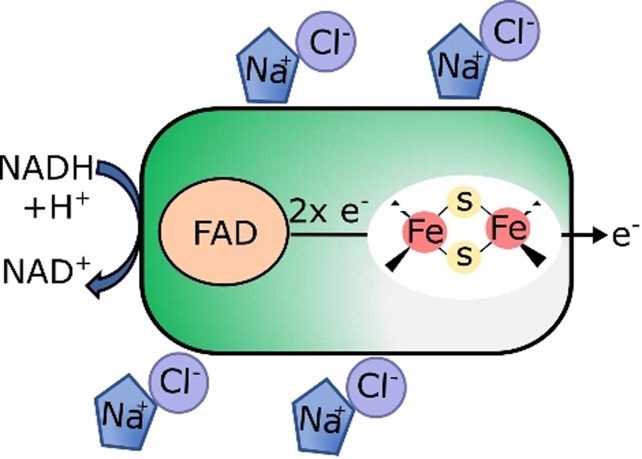

## Introduction

**Figure 1:**
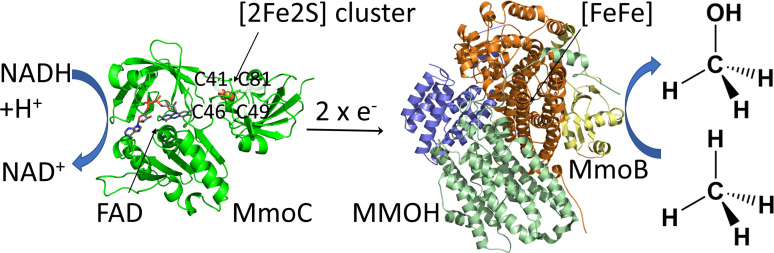
NADH dependent electron transfer by the reductase MmoC to the hydroxylase component of the soluble methane monooxygenase. The NADH dependent reductase MmoC from *M. methanica* MC09 (homology model based on PDB: 1KRH.1) and the monomeric MMOH‐MmoB complex from *Methylococcus capsulatus* (Bath) (crystal structure, PDB: 4GAM) are shown. The co‐substrate O_2_, protons and formation of water are excluded from the reaction equation on the right side. The [2Fe2S] cluster coordinating predicted cysteines are indicated.

Methane is an important energy source and has the potential to serve as a building block for biotechnological approaches.^[1]^ In nature, the soluble methane monooxygenase (sMMO) catalyses the NADH‐dependent hydroxylation of the non‐activated C−H bound of methane to methanol, which is a challenging task for organic synthesis.^[2]^ The sMMO consists of three components, including the catalytically active hydroxylase (MMOH), the NADH‐dependent reductase (MmoC), and a regulatory protein (MmoB) (Figure 1).[^3^, ^4^] The MMOH itself is an homodimer, which harbours the carboxylate‐bridged diiron centre.^[3]^ The sMMO‐specific reductase MmoC transfers two electrons from NADH via FAD and an iron‐sulfur cluster to the active site of MMOH (Figure 1)^[5]^ similar to the reductase together with the ferredoxin of most P450 monooxygenases.^[6]^ MmoC contains three domains, each binding a redox cofactor: NADH, FAD and the iron‐sulfur cluster, respectively. The FAD and NADH domains are similar to members of the ferredoxin: NADPH reductase superfamily, and the [2Fe2S] cluster domain is similar to that of plant ferredoxins (Figure 1, left).[^5^, ^7^] The regulatory component MmoB has no prosthetic group (Figure 1, right side). It modulates the access of gases to the active centre of MMOH by binding in a successive choreography together with MmoC to MMOH close to its active site and is necessary for full activity of sMMO.^[8]^ Recent crystal structures and X‐ray free electron laser structures of MMOH complexes revealed a detailed picture of the effects of MmoB and the potential inhibitor MmoD on MMOH.[^9^, ^10^] This also includes a new substrate pathway (CH_4_ and O_2_) to the diiron active site.^[11]^

The key reaction cycle intermediate of MMOH is termed “compound Q” with a unique Fe(IV)_2_O_2_ core – that is capable of breaking the exceptionally strong C−H bond (105 kcal×mol^−1^) of methane.^[12]^ The current reaction model proposes a hydrogen atom abstraction reaction to yield a bound hydroxyl radical and a methyl radical intermediate. Subsequent recombination of the radicals yields the methanol product.^[13]^ In addition to methane, sMMOs can hydroxylate a variety of molecules, e. g. halogenated aliphatic compounds such as trichloroethylene (TCE, considered as contamination in drinking water) or even aromatic compounds such as naphthalene,^[14]^ which makes it also attractive for fine chemicals synthesis.

Halotolerant enzymes have gained growing interest due to potential application under harsh conditions of industrial production processes such as high salt concentrations, temperature and presence of organic solvents.[^15^, ^16^, ^17^] Biocatalysts with habitat‐related features can be found e. g. in marine ecosystems. Here, we investigated the reductase component of sMMO from the marine methanotroph *Methylomonas methanica* MC09 (*Mm*MmoC). The *M. methanica* MC09 is a mesophilic and halotolerant methanotroph that belongs to the γ‐proteobacteria, which was isolated from the coast of Penarth, United Kingdom, and its genome was fully sequenced.^[18]^ In this work, we have successfully produced *Mm*MmoC recombinantly in *E. coli* and purified it via affinity chromatography to homogeneity. We used biochemical and spectroscopic methods to probe details of cofactor content, activity optima, kinetic properties, ROS production and redox reactions. Results are interpreted alongside the ROS significance, and the characteristics of halostable enzymes.

## Results and Discussion

**Figure 2:**
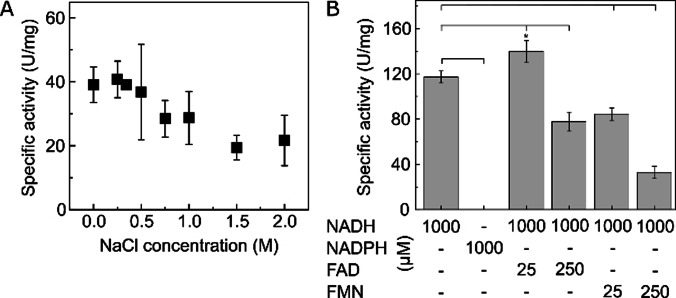
Halotolerance and FMN/FAD reconstitution experiments of soluble methane monooxygenase reductase from *Methylomonas methanica* MC09. A: Evaluation of salt tolerance for NADH‐mediated reduction of benzyl viologen by MmoC at non‐optima conditions (23 °C and pH 7.0). B: NADH specificity (clamp below) and FAD/FMN reconstitution experiments (clamp centre/top) at optima reaction conditions (0.25 M NaCl, pH 7.2 and 36 °C) The means of three technical replicates and standard deviations are shown. The asterisk indicates significance between NADH without and with 25 μM FAD (p value<0.05).

For heterologous overproduction of the flavoprotein *Mm*MmoC in *E. coli* and its subsequent purification, the *mmoC* gene was codon‐optimised and equipped with N‐terminal 10×His‐tag‐encoding sequence. The resulting plasmid pDZ02 was transferred to an E. coli chaperon co‐producing host cell strain for improved enzyme folding and yield.[19] The recombinant *E. coli* strain was grown in rich TB medium and enzyme production were initiated at low temperature. The His*‐tagged* MmoC was purified to homogeneity from the soluble cell extract by means of Ni‐NTA affinity chromatography. From 1 g cells (wet weight), we routinely obtained 16 mg of MmoC with high activity and purity (Figure 2 and Figure S2, respectively).

Considering that *M. methanica* is a halotolerant organism, we determined the NADH: benzyl viologen (BV) oxidoreductase activity under anaerobic conditions at different salt concentrations ranging from 0 to 2.0 M NaCl. The *Mm*MmoC showed activity in the whole range of tested salt concentrations. The highest activity was obtained between 0–0.5 M NaCl (Figure 2A), which is in good agreement with the physiological conditions in the marine habitat (∼0.34 M) of *M. methanica*. However, the activity declined fast at high salt concentrations (≥1.5 M NaCl), which is an indication for protein instability. But still at high salt concentrations of up to 2 M, the activity was only lowered by 50 %.

The so far described MmoCs were not evaluated for halotolerance. In most of the cases, no salt or not more than 10 mM NaCl was applied in the activity assays.[^4^, ^20^, ^21^, ^22^] Based on our homology model of *Mm*MmoC (Figure S4), we investigated whether the *Mm*MmoC has any conspicuous features related to halostable proteins. Halostable proteins are usually characterized by a higher amount of acidic amino acids at the surface to form a secured hydration shell as well as an extensive network of salt bridges inside the protein.^[23]^ Indeed, 31 glutamates and 12 aspartates were identified proposed on the surface and within the enzyme based on a homology model, respectively (Figure S3, Figure S4) representing 43–45 % more negative charged amino acids in comparison to MmoCs from selected methanotrophs (Figure S3).

Encouraged by the halotolerance, we determined the optimum temperature and pH, which were 36 °C and pH 7.2 (Figure S5). At these optimum conditions, the MmoC‐ related turnover rate or the NADH : BV oxidoreductase activity was 4585±118 min^−1^ with an apparent molecular weight of 37.9 kDa (Figure S2). The K_M_ value for NADH was calculated to be 11.6±3.48 μM (Figure S6), which is in the same range of other well‐known MmoCs.[^4^, ^24^] Similar to these, the *Mm*MmoC prefers NADH instead of NADPH. No NADPH oxidation activity could be determined at 1 mM (Figure 2B). Assuming that the NAD^+^/NADH ratio in *M. methanica* MC09 is approximately 10 : 1 and the NADH concentration is around 80 μM, as previously determined for *E. coli*,^[25]^ catalytic NADH oxidation by *Mm*MmoC will be under physiological conditions and the high affinity towards NADH close to v_max_.

Interesting, the turnover rate was a magnitude higher than MmoC from *M. capsulatus* (Bath),^[26]^ and three times higher than the related benzoate oxygenase reductase from *Pseudomonas aeruginosa* ^[27]^ with both dichlorophenylindophenol (DCIP) as the one electron acceptor. Considering the number of transferred electrons from the two‐electron donor NADH to the one‐electron acceptor benzyl viologen in case of MmoC and to the two‐electron acceptor DCIP, the MmoC activity is in the same range of benzoate oxygenase reductase, but still five times higher than MmoC from *M. capsulatus* (Bath), demonstrating that *Mm*MmoC is an attractive candidate for biotechnological approaches in combination with sMMO.

Inductive coupled plasma optical emission spectrometry (ICP‐OES) and photometric fluorescence measurements were conducted to determine the metal and organic cofactor composition of the purified MmoC, respectively. The FAD content was calculated to be 0.34–0.35 per MmoC unit. Considering the predicted presence of one [2Fe2S] cluster from the amino acid sequence alignment, MmoC showed an overall iron saturation of 36 %, when quantifying the amount of protein‐bound iron. The enzyme was shown to be not completely loaded with both cofactors. Thus, we performed reconstitution experiments with either FAD or FMN. The specific activity of MmoC was increased significantly by 20 % in presence of 25 μM FAD (Figure 2B), whereas addition of FMN did not improv activity (Figure 2B) confirming FAD as the prosthetic group. One explanation of the decreased specific activity at either 250 μM FMN or FAD could be competitive inhibition by blocking the binding site for NADH. Chemically or biochemically reconstitution of the iron sulfur cluster requires typically reducing conditions,^[28]^ which later causes loss of flavins and were thus not applied for MmoC.

**Figure 3:**
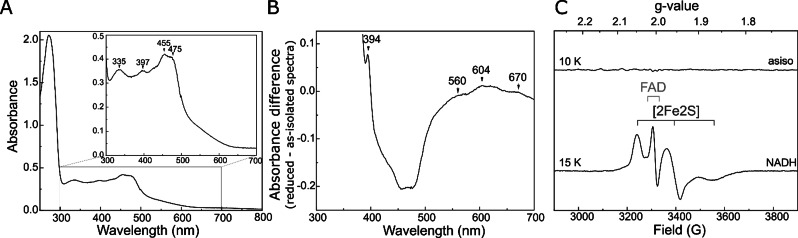
UV/vis and EPR spectra of as‐isolated and reduced *Mm*MmoC. A: UV/vis spectrum of the as‐isolated sample at 26 μM. B: Difference spectrum of the as‐isolated‐*minus*‐dithionite reduced sample; C: X‐band EPR spectra of as‐isolated (top) and NADH reduced (bottom) MmoC at 50 μM.

The content and redox activity of cofactors in MmoC was further analysed by UV/vis spectroscopy. The UV/vis spectrum of the as‐isolated (oxidized) MmoC exhibited distinct absorptions at 397 nm and 455 nm, which can be assigned to oxidized FAD (Figure 3A).^[29]^

The absorbance at 335 nm and at 475 nm were consistent with the presence of a [2Fe2S] cluster.^[28]^ The difference spectrum of sodium‐dithionite reduced and as‐isolated MmoC showed broad shoulders at 560 nm, 604 nm, and 670 nm (Figure 3B), which are typical for the neutral radical semiquinone species of FAD^[29]^ and is in line with a recent EPR study of a related MmoC.^[21]^ Moreover, a distinct peak at 394 nm appeared, which could be an indication of a lower amount of the anionic radical semiquinone species.[^29^, ^30^, ^31^, ^32^] The high similarity of the absorbance spectrum of *Mm*MmoC to that of the reductase from *M. capsulatus* (Bath)^[33]^ suggests that these proteins have the same cofactor composition with one FAD and one [2Fe2S] cluster.

Using an extinction coefficient of 12,500 M^−1^ cm^−1^ for FAD at 450 nm[^34^, ^35^] and a difference extinction coefficient 3100 M^−1^ cm^−1^ between oxidized and reduced [2Fe2S] cluster at 458 nm^[36]^ including a FeS cluster loading of 37% (see above), 0.6 FAD per *Mm*MmoC protein was calculated from the difference spectrum. This shows that the FAD in *Mm*MmoC was reduced quantitatively by dithionite and reveals the discrepancy between protein determination via flavin quantification and BCA, which overestimates cysteine‐rich proteins.^[37]^

The EPR spectrum of as‐isolated *Mm*MmoC (Figure 3C) remains featureless, as the cofactors are diamagnetic under the applied conditions. Upon incubation with the mild reducing agent NADH a complex signal emerges. By performing power‐dependent saturation experiment and recording spectra at different cryogenic temperatures (Figure S8) we could identify the rhombic signature of a [2Fe2S] cluster (g_1_=2.049, g_2_=1.957 g_3_=1.867) and the isotropic signal of a semiquinone radical at g=2.003. These findings support our observations from UV/vis spectroscopy. Regardless of whether one (dithionite) or two (NADH) electrons are donated by the reducing agents, both cofactors of *Mm*MmoC were reduced. These results are in line with Kopp *et al*. 2001 for the *M. capsulatus* MmoC^[33]^ and support the electron transmission model, namely a stepwise transfer of the two electrons from NADH to the MMOH, including one electron stored at FAD and one at the [2Fe2S] cluster.

One major side reaction of flavoenzymes is the oxidative uncoupling with molecular oxygen forming reactive oxygen species (ROS).[^38^, ^39^] In the absence of BV and in the presence of O_2_, the NADH‐mediated ROS formation activity of *Mm*MmoC was 6.0 min^−1^ (Table 1). Thus, the *Mm*MmoC‐mediated NADH oxidation towards O_2_ reduction activity was ∼400 times lower than the anaerobic BV reduction activity considering quantity of transferred electrons (Table 1). For quantification of one‐electron reduction of O_2_ and thus formation of superoxide production, we used an established assay that exploits the superoxide‐dependent oxidation of hydroxylamine to nitrite.^[34]^ The *Mm*MmoC generated 3.5 nmol superoxide per mg per min at ambient O_2_ (300 μM) corresponding to a turnover rate of 0.13 min^−1^ (Table 1).

**Table 1:** ROS formation in comparison to NADH:BV activity of *Mm*MmoC.

NADH : O_2_ [min^−1^]^[a]^	O_2_^−^ production [min^−1^]^[b]^	H_2_O_2_ production [min^−1^]	NADH : BV [min^−1^]^[c]^
6.0±2.1	0.13±0.04	5.3±2.6	4585±118

[a] NADH oxidation was measured, which is a two‐electron transferring step. [b] Superoxide production from O_2_ represents an one‐electron transfer step. [c] Benzyl viologen reduction was followed under anaerobic conditions, which represents also an one‐electron transferring step. Thus, for comparison to NADH:O_2_ specific activity in terms of transferred electrons, the NADH:BV and O_2_^−^ production activities have to be dived by factor two.

The superoxide production activity of *Mm*MmoC was more than two order of magnitude lower than the aerobic NADH oxidation activity (Table 1). This implies that ROS other than superoxide represent the main products released upon NADH oxidation/O_2_ reduction. Therefore, we analysed the capacity of the *Mm*MmoC to produce hydrogen peroxide, which is generated by a two‐electron reduction of dioxygen. H_2_O_2_ production was quantified by the horseradish peroxidase‐mediated conversion of 4‐aminoantipyrine and dichlorohydroxybenzenesulfonic acid.[^40^, ^41^] The hydrogen peroxide production activity of *Mm*MmoC was 0.14 U/mg, which is in a similar range to the MmoC from *M. capsulatus* (Bath).^[42]^ This value corresponds to a turnover rate of 5.2 min^−1^ (Table 1). In this respect, it is important to mention that superoxide decomposes spontaneously into oxygen and hydrogen peroxide.^[43]^ Interestingly, the *Mm*MmoC displayed a similar NADH oxidation activity to hydrogen peroxide production, which are both 2‐electron transfer reactions. This revealed that the hydrogen peroxide is the major ROS in MmoC‐mediated O_2_ reduction in the absence of an artificial electron acceptor or the physiological counterpart MMOH.

Together with superoxide formation, 90 % of the NADH mediated electrons were recovered in the product. Similar to the peripheral arm of mitochondrial complex I,^[44]^ MmoC has been shown to release hydrogen peroxide during catalysis in the presence of O_2_.^[45]^ In fact, ROS production by Complex I is the major origin of cellular oxidative stress, has an impact on enzyme stability and is the cause of many human diseases.^[46]^ In this study, we provide experimental evidence that *Mm*MmoC mainly generates hydrogen peroxide as catalytic by‐products during NADH conversion in the presence of O_2_, which has to be considered for biotechnological applications by adding catalase. Future interaction studies with the sMMO components MmoB and MMOH from *M. methanica* MC09 will elucidate uncoupled turnover reactions during methane conversion.

In conclusion, the sMMO reductase from the marine *M. methanica* MC09 represents a robust enzyme at various conditions especially at higher salt concentration. We revealed one flavin adenine dinucleotide (FAD) and one [2Fe2S] cluster as functional cofactors of the enzyme. The biochemical and spectroscopic characterization of this halotolerant reductase is an important step for future recombinant production of a highly interesting halostable sMMO and biotechnological methane utilisation as an attractive C1 compound.

## Conflict of interest

The authors declare no conflict of interest.

1

## Biographical Information

*Lars Lauterbach studied Technical Biology at the University of Stuttgart. In 2008, he received his PhD in microbiology from the Humboldt‐Universität zu Berlin under the supervision of Prof. Dr. Bärbel Friedrich. After research stays at UC Davis (USA) with Prof. Stephen Cramer and at University of Oxford (UK) with Prof. Kylie Vincent, he completed his postdoctoral lecture qualification with Dr. Oliver Lenz at the Technische Universität Berlin (Germany). In 2021, he was appointed as Professor in Synthetic Microbiology at the Institute of Applied Microbiology of the RWTH Aachen University. His research focuses on the design of biorefineries for the utilization and production of sustainable biofuels*.

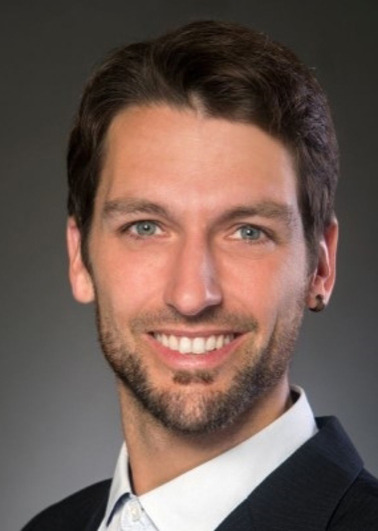

## Supporting information

As a service to our authors and readers, this journal provides supporting information supplied by the authors. Such materials are peer reviewed and may be re‐organized for online delivery, but are not copy‐edited or typeset. Technical support issues arising from supporting information (other than missing files) should be addressed to the authors.

Supporting Information

## Data Availability

The data that support the findings of this study are available from the corresponding author upon reasonable request.
