# Supplementary material for: Catalytic and Spectroscopic Properties of the Halotolerant Soluble Methane Monooxygenase Reductase from Methylomonas methanica MC09
Source: Chembiochem. 2022 Jan 13;23(5):e202100592. doi: 10.1002/cbic.202100592 (PMC9305295; doi:10.1002/cbic.202100592)
Supplement: Supplementary file 1 — Supporting Information [file CBIC-23-0-s001.pdf]

ChemBioChem

Supporting Information

Catalytic and Spectroscopic Properties of the Halotolerant Soluble Methane Monooxygenase Reductase from *Methylomonas methanica* MC09

Elisabeth Lettau[+], Domenic Zill[+], Marta Späth, Christian Lorent, Praveen K. Singh, and Lars Lauterbach*

Author Contributions

E.L. Conceptualization:Equal; Formal analysis:Equal; Methodology:Equal; Supervision:Equal; Writing – original draft:Equal
D.Z. Data curation:Equal; Methodology:Lead; Writing – original draft:Supporting
M.S. Methodology:Supporting
C.L. Formal analysis:Supporting; Methodology:Supporting
P.S. Writing – original draft:Supporting; Writing – review & editing:Equal
L.L. Funding acquisition:Lead; Project administration:Lead; Resources:Lead; Supervision:Lead; Writing – original draft:Equal; Writing – review & editing:Equal

Plasmid construction

For heterologous overproduction and subsequent purification, the *mmoC* gene from *Methylomonas methanica* MC09 was codon optimised for *E. coli* and inserted via the *Nde*I and *Bam*HI restriction sites in the plasmid pET-16b. The 5´ end of *mmoC* was equipped with a 10xHis-tag-encoding sequence resulting in the plasmid pZD02 (Fig. S1).

MmoC production and purification

E . coli BL21 with the plasmids pBB540 + pBB550 for the co-production of the chaperons DnaK, DnaJ, GrpE, ClpB, GroESL and pZD02 were grown in rich Terrific Broth (TB) medium at 37 °C until OD_{600nm} of 2 and were then induced with 0.1 mM IPTG. The protein production phase was performed at 18 °C for 14 h. The harvested cells were resuspended in twice their volume of resuspension buffer (50 mM K-PO_4, pH 7.2, 500 mM NaCl containing additional Protease Inhibitor (EDTA-free, Roche) and DNase I). After two passages through a chilled French pressure cell at 6.2 MPa, the suspension was centrifuged at 100,000× g for 45 min. The soluble extract was applied to a 2 mL Ni-NTA affinity chromatography column, washed with 6 mL of resuspension buffer without protease inhibitor and DNase I and eluted with the same buffer containing 500m mM imidazole. The eluate was then concentrated in an Amicon Ultra-15 centrifugal cell (10 kDa membrane; Amicon, Witten, Germany).

We isolated in average 16.2 mg (average of in total four purifications) homogenous MmoC from 1 g cell pellet (wet weight). In order to monitor the purification, samples of every purification step were analysed by SDS-PAGE (Fig S2). In the elution fraction, a pronounced band was detected, which correspond to the MmoC calculated size of 37.9 kDa. A second thin band of around 70 kDa most likely correspond to the co-produced chaperon DnaK.[1] Because of the already high purity of MmoC (>95%), we decided to not proceed with further purification steps. The pronounced MmoC band in the cell extract in comparison to the soluble extract indicated that a part of the produced MmoC is accumulated in inclusion bodies. The yield of proteins resulting in inclusion bodies could be increased by adding an in vitro refolding step to the purification protocol of *E. coli* BL21 containing pBB540 + pBB550 [2] . As we already achieved high yields with our existing protocol, we did not apply an *in vitro* refolding step to the purification protocol.[1] To remove imidazole after Ni-NTA affinity chromatography, the elution buffer of MmoC was exchanged *via* an Amicon Ultra-15 centrifugal filter (10 kDa membrane) to 50 mM potassium phosphate buffer pH 7.5 with 5% glycerol. Protein concentrations were determined with BCA protein assay kit (Pierce, USA) as described previously[3].

Spectroscopic measurements

Samples' UV/visible spectra were recorded with a Varian Cary 300 instrument at 16°C. The final working concentration of protein samples was 26 µM. The MmoC was reduced with 2 mM sodium dithionite under anaerobic conditions.

Electron Paramagnetic Resonance (EPR) spectroscopic experiments were performed on a Brucker EMX plus X-Band spectrometer equipped with an ER 4122 super-high Q (SHQE) resonator (Bruker Corporation) and an Oxford ESR900 helium flow cryostat (Oxford Instruments). During the measurements, an Oxford ITC4 (Oxford Instruments) temperature controller was used for adjusting the temperature. Baseline correction was performed by the subtraction of a reference spectrum obtained from a sample of buffer solution recorded with the same experimental parameters. For subsequent corrections a polynomial or spline function was used. Experimental parameters used was: 1 mW microwave power, microwave frequency 9.29 GHz, modulation amplitude 10 G and 100 kHz modulation frequency. The oxidized and NADH-reduced MmoC samples for EPR spectroscopy were analysed with a concentration of 50 µM in a volume of 100 µL.

Determination of FAD saturation

Flavin adenin dinucleotide (FAD) concentrations in MmoC were analysed photometrically in a plate reader (SpectraMax 340PC-384) at 450 nm wavelength as described previously.[4] Briefly, protein samples (10 and 3 mg/mL) were denatured by mixing with 20% trichloroacetic acid (TCA) in 1:1 ratio for 10 min at 4°C. After precipitation, the mixture was neutralised with half of the volume of the protein samples. The maintained solution was used for double determination and was filled up to 200 µL. FAD (95%) was used to prepare standards.

Optical emission spectroscopy

For the determination of iron in MmoC, metal analysis was performed using a Perkin-Elmer Optima 2100DV inductively coupled plasma-optical emission spectrometer (Perkin-Elmer, Fremont, CA, USA) following the protocol described previously[5]. In short, 500 µL of protein samples were incubated overnight with equal amount of 65% nitric acid (Suprapur, Merck KGaA, Darmstadt, Germany) at 100 °C. Samples were filled up to 5 mL with water prior to ICP-OES analysis. Buffer samples without protein were treated the same way to check if footprint of metal is dissolved in the buffer. As reference, the multielement standard solution XVI (Merck) was used.

Activity measurements

The NADH oxidation activity was measured under anaerobic conditions in a N_2-saturated activity buffer (50 mM K-PO_4 buffer pH 7.2, 0.25 M NaCl, 5 mM benzyl viologen, 1 mM NADH, ca. 100 µM sodium dithionite) similar to published literature[4]. The reaction was started by adding 2.5 µL purified MmoC (0,1 µM) to the buffer, and reduction of benzyl viologen was followed at 578 nm (VARIAN Cary 50 BIO UV-Visible Spectrometer, ε=8.9 mM-1 cm-1). For the determination of reaction optima, first of all, the salt and then the pH optima were determined at 20 °C (RT). At the end, the determination of the temperature optimum was performed. Salt concentrations were analysed in range of 0-2 M NaCl (due to marine habitat from *M. methylomonas*), pH values ranged from 6.0-8.5, and temperature studies were performed from 10-50 °C. Each measurement was performed with technical triplicates. Activity determination of MmoC with added FAD/ FMN for reconstitution investigations were measured with a biological replicate but also with technical triplicates.

Peroxide detection assay

A spectrophotometric assay previously described by Fredrico *et al* 1997.[6] was adopted and modified for the detection of hydrogen peroxide produced by MmoC. The assay is described as follows: A reaction mixture of 5.5 µL of NADH (final concentration 275 µM), 2.5-3.0 µL of purified MmoC (final concentration 2 µM) and 1 mL of 50 mM Tris-HCl buffer pH 7.2 was prepared in 1.5 mL Eppendorf tube. This reaction mixture was incubated at 30 °C for up to 5 min in a shaking thermoblock. The reaction was stopped by adding 1 ml of 3% trichloroacetic acid. The reaction mixture was centrifuged at 13,000xg for 5 min and supernatant was transferred to a new tube which was further neutralized with 5 M NaOH to pH 7.4-8. The colorless neutral solution (2 mL) was transferred to a 5 mL glass cuvette. Then, 50 µL each of AAP (final concentration 0.5 mM) and DCHBS (final concentration 5 mM), and 3 µL HRP (final concentration 4 U/mL) were added and properly mixed by inverting the cuvette three times. The formation of the pink dye was monitored spectroscopically at 515 nm ($ε515$ = 26000 M^{-1}*cm^{-1}) using Cary 50 (Varian). A negative control without HRP was used. For H_2O_2-concentration determination, a calibration line was plotted with the known amount of hydrogen peroxide (range used between 0 to 80 µM).

Superoxide assay

The formation of superoxide was indirectly detected by the formation of nitrite as described previously[7]. In this process, hydroxylamine reacts with the in-situ generated superoxide to form nitrite, which was detected in the second step by the lung reagent consisting of α-naphthylamine and sulfanilic acid. The following protocol is modified from Schneider and Schlegel[7] : For the measurement of superoxide, the reaction mixture (total volume 500 µL) consisting of MmoC (2.5 µL, 2-3 µM), NADH (10 µL, 50 mM), TCEP (5 µL, 1 mM), and hydroxylamine (5 µL, 1 mM) in Tris-HCl buffer (50 mM pH 7.2) was incubated at 30 °C in a thermoblock. After 20 min, the reaction was stopped by adding 500 µL each of α-naphthylamine (2.33 mM) and sulfanilic acid (6.33 mM). The reaction mixture was allowed to stand for additional 20 min at room temperature and then absorbance of the formed dye was measured at 530 nm using the Varian Cary 50.

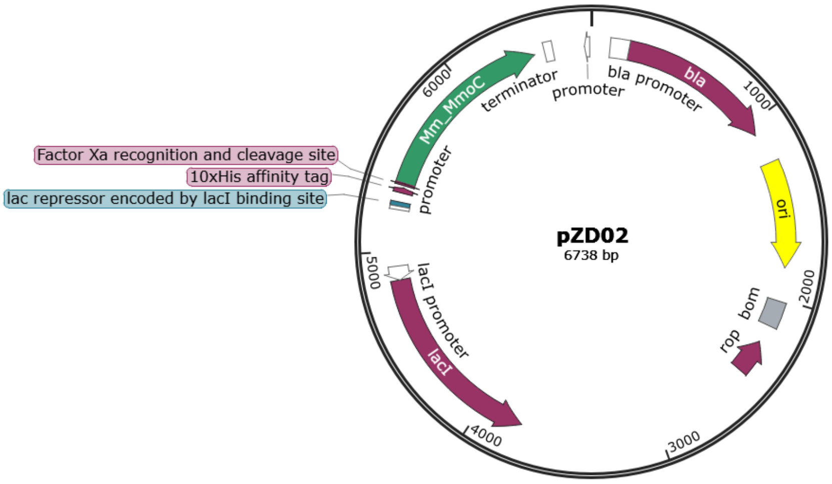

Figure S1. Plasmid map of pZD02 used for overproduction of MmoC from *M. methanica* MC09 (generated with SnapGene software, GSL Biotech, USA)

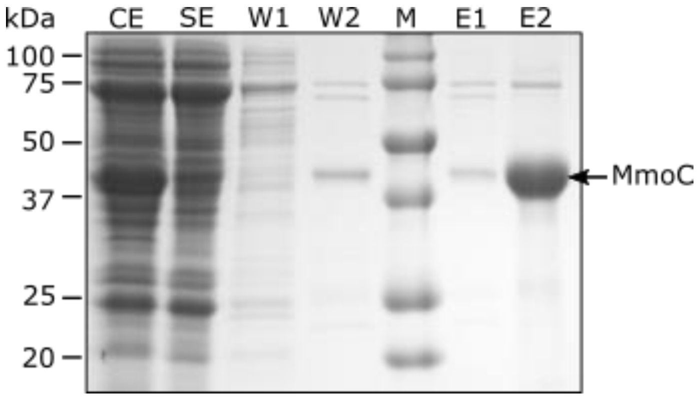

Figure S2. Purification of heterologous produced MmoC. Cell extract (CE), soluble extract (SE), the two wash fractions (W1, W2) and the elution fractions (E1, E2) were separated by SDS-PAGE and stained with Coomassie brilliant blue. A standard protein ladder (M) was used for size determination and the corresponding sizes in kDa are indicated on the left side. The arrow indicates the MmoC at 37 kDa. For E2 and for CE/SE, 2 µg and 20 µg (total protein) were applied, respectively. For W1, W2 and E1, 15 µl samples (max. pocket volume) were added.

```
MspM            ---MYQIVIETEDGETCSFECGPSEDVISAGLRQSVILLASCRAGACATCKADCTDGDYE    57
MtOB3b          ---MYQIVIETEDGETCRRM-RPSEDWISRAEA-ERNLLASCR-AGCATCKADCTDGDYE    54
MspGYJ3         ---MYQIVIETEDGETCSFECGPSEDVISAGLRQSVILLASCRAGGCATCKADCTDGDYE    57
McBath          MQRVHTITAVTEDGESLRFECRSDEDVITAALRQNIFLMSSCREGGCATCKALCSEGDYD    60
MmMC09          -MSAHLIKIVTQEGDSVCFDCFEDEDIVSAGLRQEIYLMTSCREGGCATCKGLCTDGDYE    59
MmHT12          MTSTHQVTIVTEDHESITFDCRSDEDVITAAVRQDIYLMSSCREGGCATCKGYCSEGDYV    60
                    : :   *:: ::       .** :: .   .  *::*** ..*****. *::***

MspM            LIDVKVQALPPDEEEDGKVLLCRTFPRSDLHVVVPYTYDRISFQAIQTNWLAEITECDRV    117
MtOB3b          LIDVKVQAVPPDEEEDGKVLLCRTFPRSDLHLLVPYTYDRISFEAIQTNWLAEILACDRV    114
MspGYJ3         LIDVKVQALPPDEEEDGKVLLCRTFPRSDLHLLVPYTYDRISFEAIQTNWLAEILACDRV    117
McBath          LKGCSVQALPPEEEEEGLVLLCRTYPKTDLEIELPYTHCRISFGEVG-SFEAEVVGLNWV    119
MmMC09          LGKVSSQALPTEEEENGYVLLCRCYPTSDMVVEVPYTYDRISFSPVGISVEAEIVELAKV    119
MmHT12          IGKVSAQALPSQEEEEGMVLLCRCYPTTDIEVEVPYTYERISFSPEGMDFEAEVVGLEQI    120
                :   . **:* :***:* ***** :* :*: : :***: ****     .  **:     :

MspM            SSNVVRLVLQPLTADGAAPISLNFLPGQFVDIEIPGTHTRRSYSMASVA-EDGRLEFFIR    176
MtOB3b          SSNVVRLVLQRSR-PMAARISLNFVPGQFVDIEIPGTHTRRSYSMASVA-EDGQLEFIIR    172
MspGYJ3         SSNVVRLVLQPLTADGAARISLNFVPGQFVDIEIPGTHTRRSYSMASVA-EDGQLEFFIR    176
McBath          SSNTVQFLLQKRP-DECGNRGVKFEPGQFMDLTIPGTDVSRSYSPANLPNPEGRLEFLIR    178
MmMC09          SSNVMKLQLQSPP-DE---LKIRFDAGQFFDLEIPGTTTTRSYSPANISNDRGELEFLIR    175
MmHT12          SINVVKFQLRRTG-DD---KTIKFEAGQFFDLEIPGTETTRSYSPANISNSQGDLEFLIR    176
                * *.::: *:           :.*  ***.*: **** . **** *.:    * ***:**

MspM            LLPDGAFSNYLRTQARVGQRVALRGPAGSFSLHKS-ERPRFFVAGGTGLSPVLSMIRQLH    235
MtOB3b          LLPDGAFSKFLQTEAKVGMRVDLRGPAGSFFLHDHGGRSRVFVAGGTGLSPVLSMIRQLG    232
MspGYJ3         LLPDGAFSKFLQTEAKVGMRVDLRGPAGSFFLHDHGGRSRVFVAGGTGLSPVLSMIRQLG    236
McBath          VLPEGRFSDYLRNDARVGQVLSVKGPLGVFGLKERGMAPRYFVAGGTGLAPVVSMVRQMQ    238
MmMC09          IVDNGKFSGWLQNQAHVGQKINVKGPSGIFGLKENGFTPRYFVAGGTGLAPILSMVRRMR    235
MmHT12          IVDGGKFSEFLKKEAKVGQRLKAKGPSGVFGLKENGFTPRYFVAGGTGLAPILSMVRHMK    236
                ::  * ** :*:.:*:**  :  :** * * *:.     * ********:*::**:*::

MspM            KESDPQPATLFFGVTNYEELFYVDELKALQHAMPSLDVQIAVVNVSEGNGVAKGTVIDLL    295
MtOB3b          KASDPSPATLLFGVTNREELFYVDELKTLAQSMPTLGVRIAVVNDDGGNGVDKGTVIDLL    292
MspGYJ3         KASDPSPATLLFGVTNREELFYVDELKTLAQSMPTLGVRIAVVNDDGGNGVDKGTVIDLL    296
McBath          EWTAPNETRIYFGVNTEPELFYIDELKSLERSMRNLTVKACVWHPSGDWEGEQGSPIDAL    298
MmMC09          EWEEPQTSIIYFGVNTEAEVFYAEELKRLESEMPNLGIRICVWKASDDWSGEKGSVVDVL    295
MmHT12          EWGEPQKCVIYFGVNTEAEIFHLDELEQLAAQMPTLELRNCVWKCSDDWHCEKGSVVDIL    296
                :   *.   : ***..  *:*: :**: *   * .* :: .* : . .    :*: :* *

MspM            QDELGRRAEKPDIYLCGPPGMIDAAFAAASSAGVPKEQVYLEKFLASG--      343
MtOB3b          RAELEKSDAKPDIYLCGPPGMIEAAFAAAATAGVPKEQVYLEKFLASG--      340
MspGYJ3         RAELEKSDAKPDIYLCGPPGMIEAAFAAAATAGVPKEQVYLEKFLASG--      344
McBath          REDLESSDANPDIYLCGPPGMIDAACELVRSRGIPGEQVFFEKFLPSGAA      348
MmMC09          RRDLQGGGVTPDLYLCGPPGMVDAVYAVCAEAGIAQNKIFLEKFLPSVS*      344
MmHT12          RRDLVETGAKPDLYLCGPPGMVDATFAVCADLGIPKERIYLEKFLPSGQ-      345
                : :*     .**:********::*.       *:  :::::**** *
```

Figure S3. Sequence alignment of MmoC. Black framed amino acids (AS) represent conserved cysteines coordinating the 2Fe2S-Cluster. Yellow framed AS indicate proposed FAD interactions[8]. Conserved glutamates and aspartates at the surface are indicated in orange. Proposed additional negative charged AS on the surface of *Mm*MC09 MmoC according to the homology model (Figure 1 and Figure S4) are shown in red. Conserved and *Mm*MC09 additional glutamates and aspartates within the enzyme are shown in green and blue, respectively. MmoC from *Methylomonas methanica* MC09 contains 45% and 43% more negative charged exposed and buried amino acids, respectively, in comparison to five MmoCs from other methanotrophs, while sharing conserved MmoC amino acids from the phylogenetic distinct thermotolerant *Methylococcus capsulatus* BATH and the soil bacterium *Methylovulum miyakonense* HT12. *Msp*M stands for *Methylocystis sp.* M, *Mt*OB3b for *Methylosinus trichosporium* OB3b, *Msp*GYJ3 for *Methylomonas sp*. GYJ3, *Mc*Bath for *Methylococcus capsulatus* str. Bath, *Mm*MC09 for *Methylomonas methanica* MC09, *Mm*HT12 for *Methylovulum miyakonense* HT12.

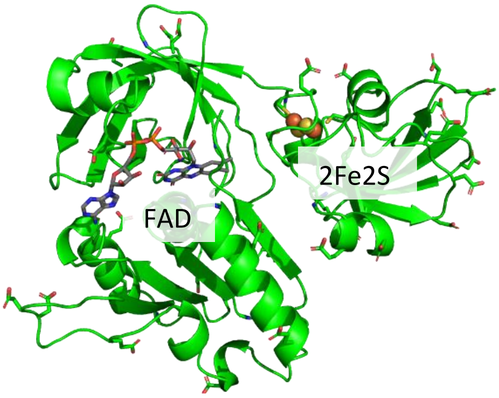

Figure S4: Homology model of MmoC from *Mm*MC09 calculated by using SWISS-MODEL[9] based on PDB: 1KRH.1 with a model quality estimation (QMEANDisCo Global) of 0.61 ± 0.05. The cofactor FAD (in stick model) and [2Fe2S] (iron and sulphur in orange and yellow spheres, respectively) were aligned from PDB 1TVC and 1JQ4. Proposed negative charged ASs on the surface are indicated.

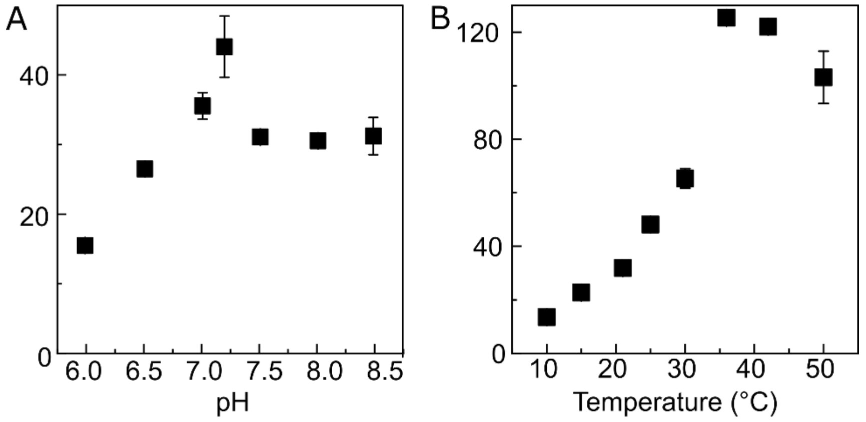

Figure S5: Catalytic optima properties. A) pH optimum was determined at 0.25 M NaCl and 23°C. B) Temperature optimum was determined at 0.25 M NaCl and pH 7.0. The means of three technical replicates and standard deviations are shown.

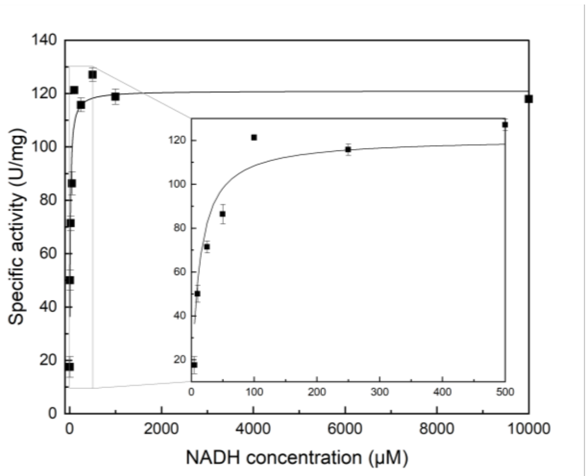

Figure S6. Michaelis-Menten kinetic of MmoC, which mediated BV reduction at different NADH concentrations. Activity was measured under anaerobic conditions at a temperature of 36°C in 50 mM K-PO_4 buffer pH 7.2 containing 250 mM NaCl and 5 mM BV. The shown values represent the means of three technical replicates of one protein preparation.

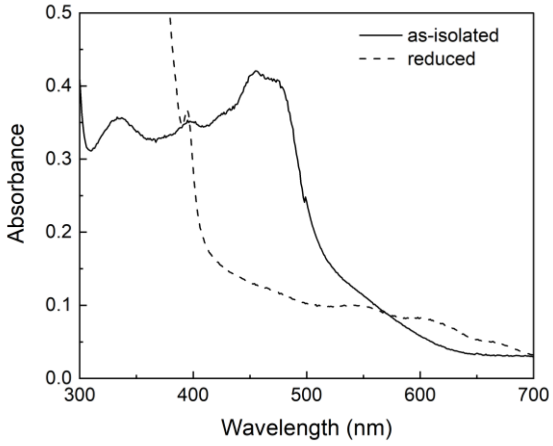

Figure S7: UV/vis of as-isolated and reduced MmoC at 1 mg / ml.

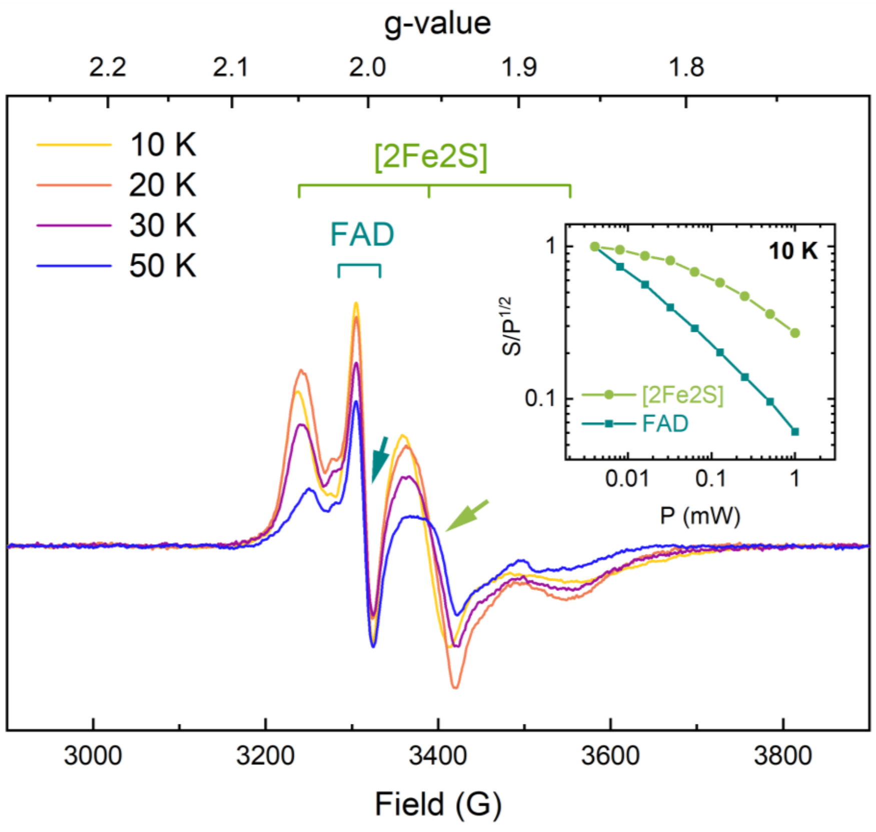

Figure S8: Temperature and microwave power dependence of the EPR signals from NADH-reduced MmoC**.** At a fixed microwave power of 1 mW and increasing temperature (T > 20 K) the rhombic signal decreases, but is still clearly visible at 50 K. This indicates the presence of a [2Fe2S] cluster rather than a [4Fe4S] cluster, as the latter is rarely visible at temperatures above 35 K due to fast spin relaxation.[10] The inset shows the power saturation of characteristic signals of FAD (g = 2.003, dark cyan arrow) and the [2Fe2S] cluster (g = 1.960, green arrow) at 10 K. Both species are fully saturated at that temperature.

References:

[1] A. de Marco, *Nature Protocols* **2007**, *2*, 2632–2639.
[2] R. Rudolph, H. Lilie, *FASEB J.* **1996**, *10*, 49–56.
[3] L. Lauterbach, O. Lenz, *JACS* **2013**, *135*, 17897–17905.
[4] L. Lauterbach, Z. Idris, K. A. Vincent, O. Lenz, *PLoS ONE* **2011**, *6*, e25939.
[5] M. Neumann, S. Leimkühler, *FEBS* **2008**, *275*, 5678–5689.
[6] R. Federico, R. Angelini, L. Ercolini, G. Venturini, A. Mattevi, P. Ascenzi, *Biochem. Biophys. Res. Commun.* **1997**, *240*, 150–152.

[7] K. Schneider, H. G. Schlegel, *Biochem. J.* **1981**, *193*, 99–107.
[8] A. Karlsson, Z. M. Beharry, D. Matthew Eby, E. D. Coulter, E. L. Neidle, D. M. Kurtz, H. Eklund, S. Ramaswamy, *J. Mol. Biol.* **2002**, *318*, 261–272.
[9] A. Waterhouse, M. Bertoni, S. Bienert, G. Studer, G. Tauriello, R. Gumienny, F. T. Heer, T. A. P. de Beer, C. Rempfer, L. Bordoli, R. Lepore, T. Schwede, *Nucleic Acids Res.* **2018**, *46*, W296–W303.
[10] S.-A. Freibert, B. D. Weiler, E. Bill, A. J. Pierik, U. Mühlenhoff, R. Lill, in *Methodes Enzymol.*, **2018**, pp. 197–226.
